# Acceptability of Early Infant Male Circumcision as an HIV Prevention Intervention in Zimbabwe: A Qualitative Perspective

**DOI:** 10.1371/journal.pone.0032475

**Published:** 2012-02-27

**Authors:** Webster Mavhu, Karin Hatzold, Susan M. Laver, Judith Sherman, Brenda R. Tengende, Collin Mangenah, Lisa F. Langhaug, Graham Hart, Frances M. Cowan

**Affiliations:** 1 Zimbabwe AIDS Prevention Project, Department of Community Medicine, University of Zimbabwe, Harare, Zimbabwe; 2 Population Services International, Harare, Zimbabwe; 3 Collaborating Centre for Operational Research and Evaluation (CCORE), UNICEF, Harare, Zimbabwe; 4 UNICEF, Harare, Zimbabwe; 5 Research Department of Infection and Population Health, Centre for Sexual Health and HIV Research, University College London, London, United Kingdom; UCL Institute of Child Health, University College London, United Kingdom

## Abstract

**Background:**

Early infant male circumcision (EIMC) is simpler, safer and more cost-effective than adult circumcision. In sub-Saharan Africa, there are concerns about acceptability of EIMC which could affect uptake. In 2009 a quantitative survey of 2,746 rural Zimbabweans (aged 18–44) indicated that 60% of women and 58% of men would be willing to have their newborn son circumcised. Willingness was associated with knowledge of HIV and male circumcision. This qualitative study was conducted to better understand this issue.

**Methods:**

In 2010, 24 group discussions were held across Zimbabwe with participants from seven ethnic groups. Additionally, key informant interviews were held with private paediatricians who offer EIMC (n = 2) plus one traditional leader. Discussions were audio-recorded, transcribed, translated into English (where necessary), coded using NVivo 8 and analysed using grounded theory principles.

**Results:**

Knowledge of the procedure was poor. Despite this, acceptability of EIMC was high among parents from most ethnic groups. Discussions suggested that fathers would make the ultimate decision regarding EIMC although mothers and extended family can have (often covert) influence. Participants' concerns centred on: safety, motive behind free service provision plus handling and disposal of the discarded foreskin. Older men from the dominant traditionally circumcising population strongly opposed EIMC, arguing that it separates circumcision from adolescent initiation, as well as allowing women (mothers) to nurse the wound, considered taboo.

**Conclusions:**

EIMC is likely to be an acceptable HIV prevention intervention for most populations in Zimbabwe, if barriers to uptake are appropriately addressed and fathers are specifically targeted by the programme.

## Introduction

Randomised trials suggest that male circumcision (MC) reduces a man's risk of acquiring HIV through heterosexual sex by 51–60% over an 18–24 months period [1], [2], [3]. Longer-term follow up suggests that this protective effect persists beyond 24 months [4], [5]. WHO/UNAIDS has recommended rapid scale-up of MC in high HIV prevalence countries to maximise intervention effectiveness at a population level [6]. In order to ensure that this protective effect is sustained in the longer-term, WHO/UNAIDS and UNICEF also recommend early infant male circumcision (EIMC) be implemented in parallel [6], [7].

Although EIMC's effects on HIV will take longer to realize, infant circumcision is likely to ultimately be more effective at preventing HIV acquisition than adult MC as the procedure is carried out long before the individual becomes sexually active, negating the risk associated with sex during the healing period [8]. Additionally, the procedure is quicker and easier to perform than adult circumcision [9]. Moreover, various surgical devices can be used to perform EIMC more simply, and these can be used by health-care cadres other than doctors, making infant circumcision potentially more accessible than adult MC. Compared to adult MC, EIMC results in fewer surgical adverse events and post-operative complications [10], [11], [12]. Furthermore, EIMC is much cheaper, with recent studies estimating that it is likely to be a cost-saving HIV prevention intervention [13], [14].

Since 2009, Zimbabwe has provided circumcision to adult and adolescent men through a collaborative effort between the government and technical agencies. The programme aims to reach 1.2 million 15–29 year-olds by 2015 [15]. Starting in mid 2012, a pilot roll-out of EIMC will be conducted. Since large-scale EIMC for HIV prevention is not yet widely available in Zimbabwe or throughout Southern Africa, there are concerns around its acceptability [8]. Clearly, acceptability will have a bearing on uptake, roll-out and subsequent effectiveness in preventing HIV.

In a 2009 representative household survey of 2,746 rural Zimbabweans (aged 18–44), 60% of women and 58% of men reported willingness to have their son circumcised; willingness was associated with increased HIV and MC knowledge [16]. Here we report on a qualitative study designed to explore these issues in more depth. Findings will inform communication strategies to promote EIMC in Zimbabwe, to plan appropriately for service delivery and to provide guidance for EIMC policy development.

## Methods

### Design and participants

The qualitative study was conducted between June and October 2010 with rural and urban participants in five of Zimbabwe's ten provinces: Bulawayo, Harare, Mashonaland West, Masvingo and Matebeleland North. Twenty-four gender-specific focus group discussions (FGDs) were held with expectant mothers (n = 6 groups), expectant fathers (n = 5 groups), grandmothers/mothers-in-law (n = 6 groups) and grandfathers/fathers-in-law (n = 7 groups) from seven ethnicities.

Key informant interviews (KIIs) were held with private paediatricians who offer EIMC (n = 2). An additional KII was held with a traditional leader from a traditionally circumcising ethnic group (Shangaan).

FGDs were conducted in either Shona or Ndebele, Zimbabwe's dominant indigenous languages, also spoken and understood by smaller ethnic groups. KIIs were conducted in English and Shona. Prior to group discussions, facilitators defined EIMC and presented basic information about the procedure, including the fact that it is quicker and safer than adult MC. Discussions then focused on issues such as perceptions of EIMC, willingness to have son undergo circumcision if it prevented HIV, barriers and motivating factors to EIMC and perceived acceptability of the intervention. Theme saturation - a situation where qualitative data collection reaches a point where no new issues emerge [17] - was reached after the 24 FGDs. Data collection was subsequently stopped. All discussions were audio-recorded.

### Analysis

Audio-recorded data were transcribed and translated verbatim into English (where necessary). Names and other personal identifiers were removed from transcripts before they were entered into NVivo 8 (QSR International, Melbourne, Australia), a qualitative data storage and retrieval program. Two researchers (CM and RBT) coded each transcription separately. Discrepancies were resolved by discussion with the senior social scientist (WM), who also independently coded all transcripts. Codes were grouped into categories and emerging themes were then identified iteratively following the general principles of grounded theory [18]. Themes and sub-themes were illustrated with verbatim quotes.

### Ethical considerations

Ethics approval was given by the Medical Research Council of Zimbabwe and the ethics board of University College London. Written informed consent was obtained on the day of the interview/discussion.

## Results

A total of 240 participants took part in FGDs; an additional three key informants were interviewed. EIMC knowledge was generally poor. Despite low knowledge, EIMC acceptability was high among participants from most ethnic groups. Older men from one traditionally circumcising population, who circumcise during adolescence, were strongly opposed to EIMC. Paediatricians reported a recent increase in parents requesting EIMC. Participants raised several concerns that have implications for circumcision roll-out. We present these themes in more depth below.

### EIMC knowledge is poor

Male circumcision knowledge in general and EIMC knowledge, in particular, is poor among the general population and especially among traditionally non-circumcising groups. Several participants, particularly (and understandably) females, did not know what the procedure involves save to say, *‘It is the removal of the skin on the penis’* (expectant mother, fgd4). When probed, they did not know how much skin is removed as well as precisely where it is removed from. Additionally, participants from traditionally non-circumcising populations were unaware of MC's benefits. ‘*We hear that it [MC] is done among the Shangaan and other people of foreign origin such as the Chewa [from Malawi] but we don't know why they do it’* (grandfather, fgd7). The same participants maintained that attempts to learn more about traditional MC have been futile since the procedure is highly secretive.

When asked to give their opinions on timing of EIMC, participants generally felt that it should be done three to six months after birth. *‘It [MC] should be done when they [babies] are about six months old. You can't do it earlier as the organ [penis] will still be too tender’* (expectant father, fgd1). Participants generally felt that fragility of the infant penis in the immediate post-partum period would result in unacceptable risk of surgical error. *‘One can easily cut off the head [penile] as well’* (father-in-law, fgd19). Private paediatricians reported parents requesting infant circumcision often when it is ‘too late’. *‘Mothers are bringing 12 month-old babies for circumcision* (HCW, KII1). Another paediatrician described challenges around circumcising toddlers. *‘As babies grow bigger they become more difficult to sedate because I do it under simple sedation…I know a lot of people want to do it under spinal anaesthesia but that is unduly traumatic to both the family and the baby’* (HCW, KII2). However, during FGDs, the feeling that it is less-risky to circumcise toddlers, as opposed to infants, was quite pervasive.

### Acceptability of EIMC is high

Despite low levels of infant MC knowledge, discussions suggested high willingness to have son circumcised in most ethnic groups – providing MC was an effective HIV prevention method. *‘Even now as I speak, if I hear that they are now circumcising children at our hospital, I will quickly take my grandchild along. I am currently faced with the burden of looking after AIDS orphans’* (grandmother, fgd9). Several participants felt that if circumcision protects one from HIV, infant and adolescent circumcision should be compulsorily offered as part of national HIV prevention efforts. *‘The government should “force” parents to circumcise their sons in the same way it “forces” them to immunise children against measles’* (grandfather, fgd20). Overall, participants felt that the impact of HIV on the younger generation is enormous, and were excited to hear that MC is partially protective.

Paediatricians reported a recent increase in the number of infant circumcisions being conducted privately as well as the number of Zimbabwean parents requesting EIMC, something previously uncommon. *‘It used to be almost entirely Muslim and Jewish parents [requesting infant MC] and then quite a number of North Africans married to Shona women but we are now getting quite a number of Zimbabweans. So the picture is definitely changing…and people often ask me about male circumcision and HIV’* (HCW, KII2). Discussions with older men who were collecting their antiretrovirals at one rural hospital corroborated quantitative findings – that HIV positive men are particularly keen to have their sons circumcised; *‘I would not want my son to also go through what I am going through now. Had circumcision come earlier, I would probably be [HIV] negative’* (father-in-law, fgd7).

### Father has ultimate decision

Study findings highlighted importance of the father in the decision-making process. *‘The man must make that decision because he is the one who knows whether or not that is practised in his clan; a woman cannot know anything about a clan to which she doesn't belong’* (father-in-law, fgd18). A female participant concurred, ‘*As the mother, I cannot decide whether or not the child should be circumcised. I will need to “sit down” [discuss] with the father and we will have to go by his decision*’ (expectant mother, fgd3). However, subsequent probing suggested that mothers-in-law/grandmothers are likely to have considerable (often covert) influence. A young woman described steps she would take if her husband refused to have their son circumcised. *‘If he [father] refuses, I will talk to his mother and she will then ask his uncles to talk to him’* (expectant mother, fgd14). EIMC decision-making is therefore likely to involve relations other than the child's parents.

### Traditionally circumcising tribes had mixed feelings

Discussions with traditionally circumcising tribes in Zimbabwe including the Xhosa, Chewa, Venda and Remba suggested that these groups are not opposed to EIMC. However, they felt that they would prefer the procedure to be performed by someone who was themselves circumcised and of the same tribe. Some Muslim participants (mostly the Chewa of Malawian origin) preferred it to be done by someone of the same religion. *‘For us to be touched [circumcised] by anyone [non-Muslim]…the truth is we don't want but we will be prepared to take our children to Indian [Muslim] doctors* (grandfather, fgd20).

However, older men from the dominant traditionally circumcising population in Zimbabwe, the Shangaan, were strongly opposed to EIMC for two reasons. Firstly, they mentioned that circumcision is just one part of a comprehensive ‘rites of passage’ ritual and should therefore not be undertaken separately. *‘We don't just circumcise. There are “lectures” that we teach those that undergo circumcision. How will we be able to teach infants?’* (grandfather, fgd6). Secondly, they noted that if infants were circumcised, their mothers would need to be involved in the process as they would nurse the wound. *‘Infants would need to be nursed by their mothers [after circumcision]. We don't want mothers to know what we do’* (traditional leader, KII3). Among the Shangaan, allowing women to see (and nurse) the MC wound is considered taboo.

### Participants' concerns

Despite high levels of acceptability, community members raised several key questions discussed below.

#### Safety of the procedure

Community members questioned the safety of EIMC. As previously stated, safety-related concerns were based on the assumption that the newborn infant's penis is too fragile to be circumcised, leading participants to feel that, to maximise safety, the procedure should only be performed by highly-trained doctors. *‘This thing should be done by doctors who really know how to do it and no one else’* (mother-in-law, fgd23). Participants were also concerned about the possibility of excessive bleeding and keloid scarring. ‘*What if my child gets swollen like those people who have a large growth from ear piercing…?’* (expectant father, fgd11).

#### Handling and disposal of removed foreskin

Customarily, Zimbabweans are worried about disposal of body fluids/tissues as they fear that these may be used by ‘witches’ to cause subsequent harm. For example, people burn shaved hair and nail clippings in case these end up in the wrong hands. With infants, disposal of the umbilical stump is a culturally-sensitive issue which involves mothers-in-law/grandmothers. Unilateral disposal of the umbilical stump by a young couple/mother can have serious implications. Community members were therefore anxious about the fate of the amputated foreskin: *‘What will happen to the piece [foreskin] that gets removed?’* (grandfather, fgd5). Another participant stated, *‘They (HCWs) should ensure that pieces that get removed are carefully disposed of so that they do not end up in the hands of those who could use them as ‘muti’ [traditional charm]* (mother-in-law, fgd18).

Some participants felt that parents should be given the foreskins in order that they would be able to dispose of them themselves, drawing parallels with the common practice of obtaining the infant umbilical stump from health-care workers. *‘The foreskin [infant] is just the same as the umbilical cord [stump]; both should be given back to the child's parents’* (father-in-law, fgd19). Mothers-in-law/grandmothers strongly articulated that should young couples decide to circumcise infants on their own, *at a minimum* elders need to be involved in foreskin disposal. *‘As is the case with the umbilical cord [stump], I should be the one who decides where and how to dispose the piece that gets remo*ved’ (mother-in-law, fgd13). Overall, older male and female participants alike felt that the infant's removed foreskin should be given to the child's relations.

#### Motivation behind free service provision

A few participants questioned why MC in general and EIMC specifically, is being or will be provided free of charge. *‘Why is this thing done for free yet operation [caesarean section] on a pregnant woman is costly?*’ (father-in-law, fgd17). These participants felt that caution should be exercised when accepting this service since it is donor-driven and the motive of the countries paying for it are unclear.

## Discussion

Data from this qualitative study corroborate some of the quantitative findings from our population-based survey, namely that EIMC was seen as widely acceptable. However, given the very low levels of knowledge or experience of EIMC, it is not clear whether this hypothetical acceptability will translate into actual acceptability once EIMC roll-out begins. It is clear though that participants were very interested in the intervention as described, that is, one that could protect their sons against future HIV.

This qualitative study additionally identified new issues which have implications for EIMC implementation. Firstly, given the low levels of knowledge about the procedure, it will be important to provide information at a community level to enhance the procedure's acceptability. These qualitative findings reinforce the need for multi-faceted awareness campaigns (e.g. community mobilisation, road shows) to ensure that everyone in the community is reached and not just those in contact with clinical services [16].

Education needs to include both women and men; it should also target multiple generations. While reinforcing the crucial role fathers play in EIMC decision-making shown elsewhere [19], [20], our data suggest that fathers need to be provided with information *directly* not just through their wives. As caretakers of their family's health, women often receive information from health-care centres during routine visits. Yet men are notoriously hard to reach via health services [21], [22], [23]. Other venues should therefore be considered. Workplaces and beer halls have been successfully used to increase knowledge of health-related matters [24], [25].

Participants raised concerns around the safety of EIMC. Similar concerns have also been observed in other studies across the region [8], [16], [26], [27]. Study participants generally thought a newborn's penis is ‘too fragile’ to undergo circumcision. Overall, the level of anxiety observed suggested that these concerns need to be addressed head-on to improve EIMC uptake. Awareness campaigns need to adequately communicate that it is not only possible but also preferable for circumcision to be done soon after birth. In practice, however, acceptability and uptake will depend on perceptions of procedure's safety [28]. Provision of EIMC will therefore need to be carefully supervised and monitored to ensure i) a good cosmetic result and ii) that adverse effects are prevented.

Participants strongly felt that safe EIMC can only be provided by highly-trained doctors. However, in practice, it is likely that EIMC will largely be performed by midwives/nurses since it is an uncomplicated procedure [8], [9]. Communication materials/models should focus on the fact that nurses and midwives have the relevant skill/expertise to perform the procedure and that including them in EIMC delivery will make the procedure accessible through more remote health-care facilities, which are only served by nursing cadres.

Study findings support the now well-recognised notion that cultural beliefs are integral to successful MC provision [6], [8], [12], [29]. In this study, several ethnic groups were concerned about handling and disposal of the removed foreskin. Secondly, some participants preferred circumcision to be performed by individuals of either the same tribe or religion. Thirdly, older Shangaan men strongly opposed EIMC as they felt that it undermines their tradition by separating circumcision from adolescent initiation, in addition to allowing women (mothers) to nurse the wound, considered taboo.

These findings have at least three implications for rolling-out circumcision, in general and EIMC, specifically. Firstly, implementers will need to recognise and understand cultural and religious beliefs attached to MC among certain groups [12]. It will be important to engage key traditional and religious leaders in efforts to mobilise a wider understanding and acceptance of circumcision for HIV prevention. Secondly, MC providers need to be drawn from diverse ethnic/religious circles. Lastly, EIMC communication materials/models should specifically address concerns around safety, timing and tissue disposal.

This study has several strengths. Firstly, this research was conducted with participants representing the majority of ethnic groups, and in half of Zimbabwe's ten provinces. Secondly, our sample size was large for a qualitative study. The sample was purposively selected to ensure a wide range of views were heard from a diverse population. Thirdly, we managed to achieve theme saturation, an important component of qualitative research.

There were some limitations to this study. Firstly, although EIMC was defined prior to group discussions, some participants still discussed neonatal circumcision and had to be reminded that they needed to focus on infant circumcision. Secondly, we explored EIMC acceptability in the absence of widely-available services or any communication campaign that specifically provides information about infant circumcision. Hypothetical acceptability may be quite different from actual acceptance when EIMC is eventually rolled-out [8]. It will be crucial to assess EIMC acceptability within the context of actual roll-out. Lastly, while religious beliefs might be expected to affect attitudes/beliefs related to EIMC, neither our qualitative nor quantitative data found any indication of this. Nonetheless, it may be worth exploring this issue in more depth with religious groups that are specifically known to resist biomedical interventions and who make up a significant minority of the population in Zimbabwe and sub-Saharan Africa more widely.

In conclusion, this study found that EIMC is a potentially acceptable HIV prevention intervention in Zimbabwe and provided a framework for addressing likely barriers to uptake. Specifically, awareness campaigns that increase knowledge will be crucial to translating hypothetical acceptability into actual uptake. These data suggest that barriers are not insurmountable - which bodes well for achieving high EIMC targets in sub-Saharan Africa, in general and Zimbabwe, specifically.
